# Inhibited Wnt Signaling Causes Age-Dependent Abnormalities in the Bone Matrix Mineralization in the Apert Syndrome FGFR2^S252W/+^ Mice

**DOI:** 10.1371/journal.pone.0112716

**Published:** 2015-02-18

**Authors:** Li Zhang, Peng Chen, Lin Chen, Tujun Weng, Shichang Zhang, Xia Zhou, Bo Zhang, Luchuan Liu

**Affiliations:** 1 Department of Stomatology, Daping Hospital & Research Institute of Surgery, Third Military Medical University, Chongqing 400042, China; 2 Department 4, Daping Hospital & Research Institute of Surgery, Third Military Medical University, State Key Laboratory of Trauma, Burns and Combined Injury, Chongqing 400042, China; 3 Neurosurgery Department, PLA 324 Hospital, Chongqing, China; Inserm U606 and University Paris Diderot, France

## Abstract

Apert syndrome (AS) is a type of autosomal dominant disease characterized by premature fusion of the cranial sutures, severe syndactyly, and other abnormalities in internal organs. Approximately 70% of AS cases are caused by a single mutation, S252W, in fibroblast growth factor receptor 2 (FGFR2). Two groups have generated FGFR2 knock-in mice *Fgfr2* ^S252W/+^ that exhibit features of AS. During the present study of AS using the *Fgfr2* ^S252W/+^ mouse model, an age-related phenotype of bone homeostasis was discovered. The long bone mass was lower in 2 month old mutant mice than in age-matched controls but higher in 5 month old mutant mice. This unusual phenotype suggested that bone marrow-derived mesenchymal stem cells (BMSCs), which are vital to maintain bone homeostasis, might be involved. BMSCs were isolated from *Fgfr2* ^S252W/+^ mice and found that S252W mutation could impair osteogenic differentiation BMSCs but enhance mineralization of more mature osteoblasts. A microarray analysis revealed that Wnt pathway inhibitors SRFP1/2/4 were up-regulated in mutant BMSCs. This work provides evidence to show that the Wnt/β-catenin pathway is inhibited in both mutant BMSCs and osteoblasts, and differentiation defects of these cells can be ameliorated by Wnt3a treatment. The present study suggested that the bone abnormalities caused by deregulation of Wnt pathway may underlie the symptoms of AS.

## Introduction

Fibroblast growth factor (FGF) and fibroblast growth factor receptor (FGFR) play critical roles in the process of osteogenesis in both embryonic development and postnatal life [1]. The FGFR family is comprised of four trans-membrane receptor tyrosine kinases (FGFR1–4) activated through induced dimerization and autophosphorylation upon FGFs binding [2], [3]. Among four FGFRs, FGFR2 mediates signals from many of the 22 fibroblast growth factors (FGF1–22) [4].

Craniosynostosis, a condition in which one or more of the fibrous sutures prematurely fuses, is a common craniofacial abnormality affecting 1 in 2,500 newborns [5], [6]. Approximately 20% of cases are attributable to gain-of-function mutations in FGF receptors (FGFRs) [7]. Apert syndrome is one of the most severe forms of craniosynostosis. It is characterized by synostosis of the coronal, sagittal, and lambdoid sutures and by other abnormalities, including severe syndactyly and defects of the internal organs [8]. Two missense mutations in adjacent amino acids, Ser252Trp and Pro253Arg, of FGFR2 together account for 99% of reported cases of Apert [9], [10]. The first mutation, S252W, was found to be involved in 67% of cases, and it is considered to be associated with more severe craniofacial anomalies, and the second mutation, P253R, may cause more severe syndactyly [11], [12]. Both mutations are located in the highly conserved region linking the immunoglobulin-like II and III domains. Both can lead to loss of ligand specificity associated with hyperactivity of FGFR2 [13], [14]. In vivo and in vitro studies have shown that S252W mutation can perturb normal bone development by affecting proliferation, differentiation, and apoptosis [15]–[18].

Activation of FGF signaling mainly leads to activation of two primary pathways, namely the mitogen-activated protein kinase (MAPK) pathway and protein kinase C (PKC) pathway [1], [2], [4], [19]. S252W mutation activates PKC pathways to promote apoptosis in human osteoblasts [16]. Suppression of the MAPK-ERK pathway by means of RNA interference or pharmacological inhibition can prevent certain skeletal abnormalities in *Fgfr2* ^S252W/+^ mouse models [20] Elevation of the phosphorylation level of AKT and p38 has been detected in calvarial tissues of newborn *Fgfr2* ^S252W/+^ mice and immortalized cells [21]. Despite these advances in the understanding of FGFR2 mutations, the molecular mechanisms underlying pathogenesis of Apert syndrome still remain poorly understood.

Two groups have created FGFR2 knock-in mutant mice, called *Fgfr2* ^S252W/+^ mice. These mice exhibit features similar to those of human Apert syndrome, and they have become popular models for investigations of the mechanism underlying Apert syndrome [22], [23]. During the course of investigating Apert syndrome using a mouse model, an age-dependent bone mass phenotype of *Fgfr2* ^S252W/+^ was observed. Results showed that 2 month old *Fgfr2* ^S252W/+^ mice have less long bone mass than age-matched wild type mice. However, 5 month old mutant mice have more bone mass than control mice, indicating defective bone homeostasis. Bone-marrow-derived mesenchymal stem cells (BMSCs) are crucial players in adult bone homeostasis. To further understand the mechanism underlying abnormal bone homeostasis at the molecular level, BMSCs from both wild type and mutant mice were isolated. Results showed that S252W mutation inhibited proliferation of BMSCs. Using in vitro osteogenic differentiation culture, early S252W mutation inhibited osteogenic differentiation of BMSCs but later promoted mineralization of more mature osteoblasts. Microarray analysis revealed increased expression of Wnt pathway inhibitors SFRP1, SFRP2, and SFRP4. A later study confirmed that Wnt/β-catenin signaling was attenuated in mutant BMSCs. Forcedly increasing Wnt/β-catenin signaling by adding Wnt3a to cell cultures was found to reverse all the proliferation and differentiation defects in mutant cells. In this way, S252W mutations were found to inhibit osteogenic differentiation of BMSCs in early stages of development but promote osteoblast maturation and mineralization in later stages by modulating Wnt/β-catenin signaling. This mechanism underlies the abnormalities observed in ossification in *Fgfr2* ^S252W/+^ mice.

## Materials and Methods

### Fgfr2^S252W^ mice

The Apert mouse model *Fgfr2^NeoS252W^* and the *EIIaCre* mice were provided by Dr. Chuxia Deng of NIH. Heterozygous mutant mice generated with *Fgfr2^NeoS252W^* and *EIIaCre* are here referred to as *Fgfr2^S252W/+^* [22]. Genotyping of tail DNA was used to distinguish mutant from wild-type progeny by PCR analysis. Mice were euthanized by cervical dislocation. *Fgfr2^S252W/+^* mice demonstrated significant craniofacial anomalies as evidenced by micro-CT studies (Fig. S1A). Two- or five-month-old *Fgfr2^S252W/+^* mice and littermate controls were used for phenotypic analyses. Mice were housed in the Daping Hospital's animal facility and all protocols were approved by the Institutional Animal Care and Use Committee of Daping Hospital.

### Alcian blue-alizarin red staining of the mouse skeleton

Mouse skeleton staining was performed as described previously [24]. Skeleton specimens were fixed in 75% ethanol for 2 h followed by fixation in 95% ethanol for 2 days and then in acetone for another 2 days. Staining solution was made by mixing 3 g/L Alcian blue solution, 1 g/L alizarin red solution, acetic acid, and 75% ethanol at a volume ratio of 1∶1∶1∶17. Skeleton specimens were stained for 24 h, washed in distilled water, soaked in 10 g/L potassium hydroxide solution for 48 h, and then stored in glycerol. Cartilage was stained blue and bone tissue was stained red.

### Micro-computed tomography (micro-CT)

Femurs were isolated from 2- and 5-month-old mice. Fixed non-demineralized femurs and the femoral cancellous bones of the distal metaphysic and the middle shaft were scanned with micro-CT (µCT-80, Scanco Medical AG, Bassersdorf, Switzerland) as reported previously [25]. Images (IMAQ) were acquired at 70 kV and 113 mA. Two-dimensional images were used to generate three-dimensional reconstructions for 3D analysis. The analysis of the specimens involved the following bone measurements: trabecular and cortical bone volume fraction (Tb.BV/TV, Ct.BV/TV, %), trabecular number (Tb.N), trabecular and cortical thickness (Tb.Th, Ct.Th), trabecular separation (Tb.Sp), trabecular structure model index (Tb.SMI), trabecular and cortical bone mineral density (Tb.BMD, Ct. BMD) [26].

### Histology and histomorphometric analysis

The tibiae were fixed in 40% ethanol overnight and dehydrated in a graded ethanol series. For analysis of parameters of bone formation, the bones were embedded in a mixture of methyl methacrylate and dibutyl phthalate. Von Kossa staining was performed to identify minerals and osteoids. Specifically, five-micron sections of proximal tibiae were stained with 2% silver nitrate for 20 min under UV light and with 0.1% toluidine blue for 1 min. The Tb.BV/TV and Tb.Sp of tibiae were analyzed using OsteoMeasure system (OsteoMetrics, U.S.). The tibiae were fixed in 4% paraformaldehyde overnight at 4°C, rinsed in PBS, and decalcified in 15% EDTA (pH 7.4) for 20–30 days. Then they were embedded in paraffin as described previously [27]. Six-micron sections were prepared for H&E staining.

### Serum biochemistry and PINP

Serum was obtained from 2-month-old mice for total Ca and phosphate analysis using routine automated techniques at the Daping Hospital Diagnostics Laboratory. The serum level of Procollagen I N-Terminal Propeptide (PINP) was examined using Mouse PINP ELISA Kit according to the manufacturer's instructions (USCNK, Wuhan, China). The absorbance of stopped reaction mixtures was measured at 450 nm. The intensity of the color was inversely proportional to the concentration of PINP.

### BMSCs isolation and culture

BMSCs were harvested from 6- to 8-week-old mice as described previously [27]. Mice were euthanized and both femurs and tibiae were aseptically removed. Then the ends of the femurs and tibiae were cut and the bone marrow was flushed out with 5 ml C57B/6 Mouse Mesenchymal Stem Cell Growth Medium containing 10% fetal bovine serum (FBS), 1% penicillin/streptomycin and 1% glutamine (Cyagen, San Francisco, CA, U.S.), here called standard medium. The cells were cultured in standard medium at 37°C in a 5% CO_2_ humidified incubator. BMSCs were allowed to adhere to the plastic support for 24 h before the first medium change. Nonadherent cells were removed by flushing with 0.1 M DPBS and the standard medium was replaced every 3 days. Cells in passage 2 were used for the experiments. For Wnt stimulation, cells were cultured in standard medium with 100 ng/ml recombinant mouse protein Wnt-3a (R&D system Inc., Minneapolis, MN, U.S.).

### Cell proliferation assay

Cell proliferation was detected using Cell Counting Kit-8 (Beyotime, Shanghai, China). BMSCs (1×10^4^ cells per well) were plated in 96-well plates. Wells containing the standard medium without cells were used as blanks. The plates were incubated for 0 day, 2 days, 4 days, 6 days, 8 days, 10 days, and 12 days. Then 20 µl CCK-8 dye solution was added and incubated for 4 h at 37°C. After 4 h of incubation, optical density D was measured on a microplate spectrophotometer (MD VersaMax, Molecular Devices, Sunnyvale, CA, U.S.) at a wavelength of 450 nm. The proliferation rate (%) was calculated.

### Adipogenic differentiation and oil red O staining

BMSCs in passage 2 were replated in the standard medium at 1×10^5^ cells/cm^2^ in 6-well plates. Cells were incubated at 37°C in a 5% CO_2_ humidified incubator. After the cells were 100% confluent, the standard medium was carefully aspirated off, and 2 ml Adipogenic Differentiation Medium A (induction medium) containing 10% FBS, 20 µg/ml insulin, 10 µM IBMX, 10 µM rosiglitazone, 10 µM dexamethasone, 1% penicillin/streptomycin, and 1% glutamine were added (Cyagen). Three days later, the Adipogenic Differentiation Medium A was replaced with Adipogenic Differentiation Medium B (maintenance medium), containing 10% FBS, 20 µg/ml insulin, 1% penicillin/streptomycin and 1% glutamine (Cyagen). Then, 24 h later, the medium was changed back to induction medium. To optimally differentiate BMSCs into adipogenic cells, the cycle of induction and maintenance was repeated three times. After three cycles, the cells were cultured in maintenance medium for an additional 7 days by replacing the medium every 3 days. After differentiation, Adipogenic Differentiation Medium was removed and the cells were rinsed twice with 0.1 M DPBS. Cells were fixed with 4% formaldehyde solution for 30 min. Then the cells were rinsed twice with 0.1 M DPBS and stained with oil red O (Cyagen) for 30 min at room temperature (RT). Cells were visualized under a light microscope and images were captured.

### Osteogenic differentiation and Alizarin red staining of mineralized osteoblast

BMSCs in passage 2 were replaced in the standard medium at 1×10^5^ cells/cm^2^ in 6-well plates. The cells were incubated at 37°C in a 5% CO_2_ humidified incubator. After the cells were approximately 80–90% confluent, the standard medium was aspirated off, and 2 ml Osteogenic Differentiation Medium containing 10% FBS, 20 µg/ml ascorbate, 10 mmol/l β-glycerophosphate, 10 µM dexamethasone, 1% penicillin/streptomycin, and 1% glutamine (Cyagen) was added. Cells were re-fed every 3 days from day 4 to day 21 using fresh Osteogenic Differentiation Medium. After 21 days of osteogenic differentiation, cells were rinsed two times with 0.1 M DPBS and fixed in 4% formaldehyde solution for 30 minutes. Then cells were washed with water and stained with 0.2% Alizarin red (Sigma-Aldrich, St. Louis, MO, U.S.) in 2% ethanol for 30 min at 37°C. After staining, cells were washed four times with water and dried at 37°C. For quantification of mineral content, bound dye was dissolved with 5% SDS, 0.5 N HCl, and measured at 415 nm [28].

### Alkaline phosphatase activity and staining

ALP activity was determined using an ALP kit (Sigma-Aldrich) according to the manufacturer's instructions. Cells were washed twice with 0.1 M DPBS, and lysed in 10 mM Tris–HCl containing 2 mM MgCl_2_ and 0.05% Triton X-100 (pH 8.2) at 4°C. The lysates were centrifuged at 12,000 g for 10 min at 4°C, and the supernatants were mixed with ALP detection buffer and incubated at 37°C for 30 min. The reaction was then stopped by the addition of 0.1 M NaOH and monitored at 405 nm. Total protein was measured spectrophotometrically using a Micro-BCA Protein Assay Kit (Pierce, Rockford, IL, U.S.) and read at 562 nm. The enzymatic activity of ALP was normalized to the total protein content of the sample (405/562 nm) [29]. For ALP staining, cells were fixed with 4% paraformaldehyde for 30 min at room temperature (RT), washed with 0.1 M DPBS, and stained with BCIP/NBT solution (Beyotime) for 30 min at RT in the dark. The alkaline-phosphatase-positive cells were stained blue and purple.

### RNA isolation and quantitative RT-PCR

Total RNA was isolated from BMSCs using Trizol reagents (Invitrogen, Carlsbad, CA, U.S.) according to the manufacturer's protocol. The sequences of the primers and the expected sizes of the PCR products are shown in Table 1. All reactions were performed in a Mx3000P PCR machine (Stratagene, La Jolla, CA, U.S.) using a One Step SYBR PrimeScript RT-PCR Kit (Takara, Shiga, Japan) according to the manufacturer's protocol. Each run was performed three times.

**Table 1:** Primer with their sequence, length of amplification (bp) used for qRT-PCR in this study.

Gene	Sense primer	Antisense primer	Size (bp)
LPL	5^′^-GGGTCACCTGGTCGAAGTAT-3^′^	5^′^-CTCTCTGCAATCACACGGAT-3^′^	122
PPARγ	5^′^-GCCTAAGTTTGAGTTTGCTGTG-3^′^	5^′^-GCGGTCTCCACTGAGAATAATG-3^′^	97
Collage I	5^′^-ACTTTGCTTCCCAGATGTCC-3^′^	5^′^-CCTTGGAAACCTTGTGGACC-3^′^	133
*oc*	5^′^-CGCTCTGTCTCTCTGACCT-3^′^	5^′^-TCACTACCTTATTGCCCTCCT-3^′^	93
*op*	5^′^-GATTCTGTGGACTCGGATGAAT-3^′^	5^′^-GTAGGGACGATTGGAGTGAAAG-3^′^	107
Runx2	5^′^-GCCACTTACCACAGAGCTATT-3^′^	5^′^-GAGGCGATCAGAGAACAAACT-3^′^	108
osterix	5^′^-GCAAATGACTACCCACCCTT-3^′^	5^′^-ACGAGCCATAGGGATGAGTC-3^′^	148
SFRP1	5^′^-CCAGTTCCAGGCTTCCTAA-3^′^	5^′^-GCTTCTGGAGCACATCTTGA-3^′^	146
SFRP2	5^′^-CAAACATTTCGTTGCTCGTT-3^′^	5^′^-TCATGCAATGAGGAATGGTT-3^′^	115
SFRP4	5^′^-GGTTGCAATGAGGTCACAAC-3^′^	5^′^-ATATGTGGACACTGGCAGGA-3^′^	116
CyclinD1	5^′^- ACCCTGACACCAATCTCCTC -3^′^	5^′^- ACCCTGACACCAATCTCCTC -3^′^	116
Lef1	5^′^- ATGGAAGCTTGTTGAAACCC -3^′^	5^′^- AAGAGGTGGCAGTGACTGTG -3^′^	128
fzd4	5^′^- GCAGATCCAAATACGTGGTG -3^′^	5^′^- TGGAGGTTCATTAGGCATCA -3^′^	115

### Western blot analysis

BMSCs were cultured in the aforementioned media for the indicated lengths of time. Proteins were extracted with a buffer containing 1% Nonidet P-40, 50 mM Tris–HCl, pH 7.4, 150 mM NaCl, 0.1% SDS supplemented with a cocktail of protease inhibitors (Roche Diagnostics Co., Indianapolis, IN) [30]. Proteins were separated on a 10% SDS-PAGE gel and transferred to the Immobilon-P PVDF membrane (Millipore, Billerica, MA, U.S.). Then the proteins were probed with antibodies specific to β-catenin, SFRP1, SFRP2, and SFRP4 (Cell Signaling, Beverly, MA, U.S.).

### Microarray

Total RNA samples were extracted from BMSCs of 6–8-week-old wild-type and mutant mice using a RNeasy Mini kit (Qiagen, Valencia, CA, U.S.). Three samples were prepared per genotype. The RNA concentration and purity were checked by OD260/OD280 (≧1.8) and OD260/OD230 (≧1.5), and the yield and quality were accessed (RIN≧6.0) using Agilent 2100 Bioanalyzer (Agilent Technologies, Santa Clara, CA, U.S.A). Fluorescent aRNA targets were prepared from 1 or 2.5 µg total RNA samples using OneArray Amino Allyl aRNA Amplification Kit (Phalanx Biotech Group, Taiwan) and Cy5 dyes (Amersham Pharmacia, Piscataway, NJ, U.S.). Fluorescent targets were hybridized to the Mouse Whole Genome OneArray v2 (Phalanx Biotech Group, Taiwan) with Phalanx hybridization buffer using Phalanx Hybridization System. After 16 h hybridization at 50°C, non-specific binding targets were washed away by three different washing steps (Wash I 42°C 5 mins; Wash II 42°C, 5 mins, 25°C 5 mins; Wash III rinse 20 times), and the slides were dried by centrifugation and scanned using an Axon 4000B scanner (Molecular Devices, Sunnyvale, CA, U.S.). The intensities of each probe were determined using GenePix 4.1 software (Molecular Devices). The raw intensity of each spot was loaded into a Rosetta Resolver System (Rosetta Biosoftware) to process data analysis. The error model of Rosetta Resolver System could remove both systematic and random errors form the data. Those probes with background signals were filtered out. Probes that passed the criteria were normalized by 50% median scaling normalization method. The technical repeat data were subjected to Pearson correlation coefficient calculation to confirm reproducibility (R value>0.975). Normalized spot intensities were transformed to gene expression log2 ratios between the control and treatment groups. The probes with log_2_ ratio ≥1 or log_2_ ratio ≤−1 and *P*-value <0.05 were defined as differential genes for further pathway enrichment analysis.

### Statistical analysis

Data were collected from at least three independent experiments, and the results are expressed as means±standard deviation (SD). Data were evaluated statistically in SPSS Windows, version 10.0. (SPSS Inc., Chicago, IL, U.S.). Statistics were assessed using Student's t-test, assuming double-sided independent variance. *P* values <0.05 were considered to be statistically significant.

## Results

### S252W mutation of FGFR2 affects bone mass

**Figure 1:**
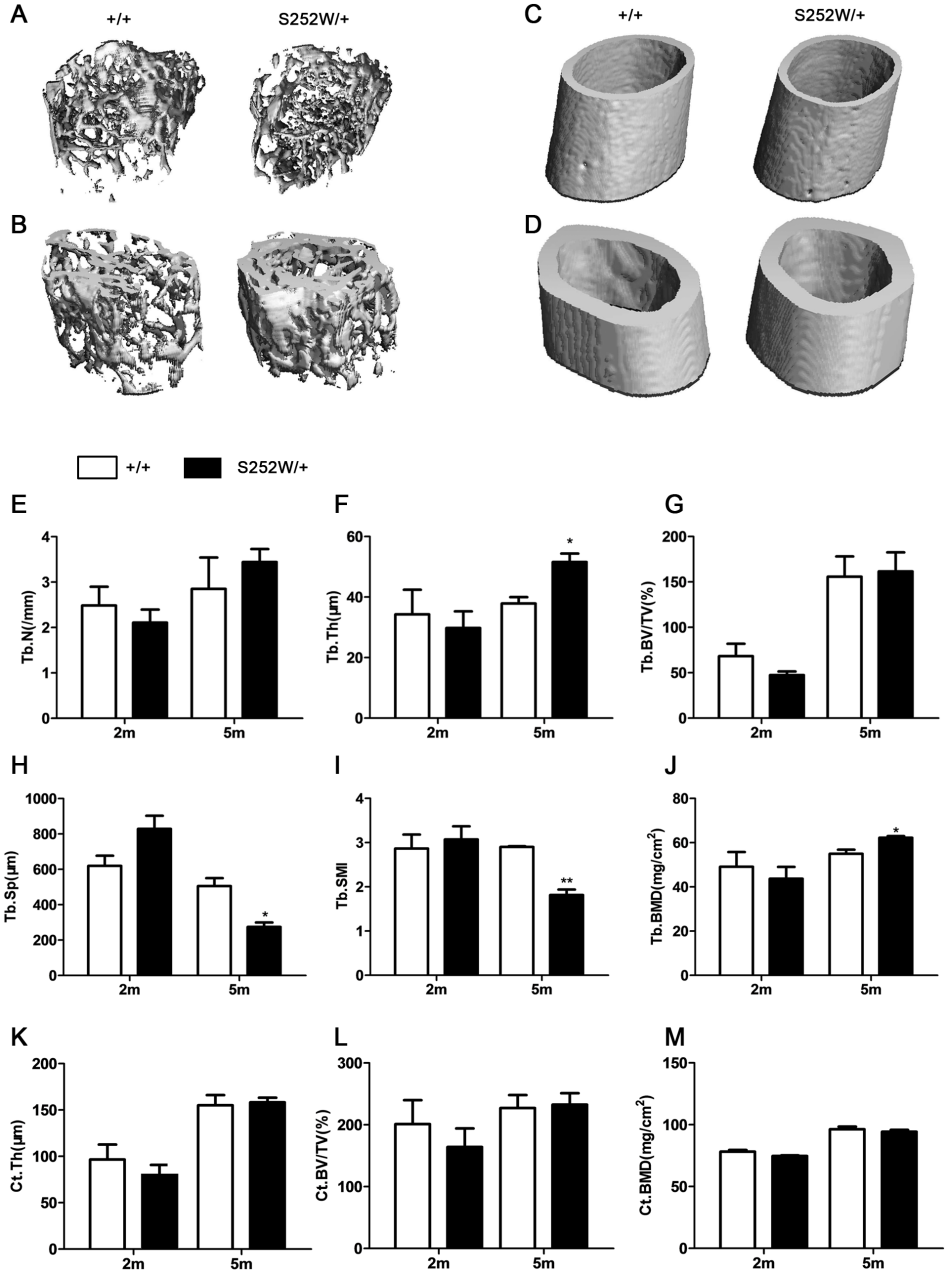
Bone abnormalities in *Fgfr2* ^S252W/+^ mice. Quantitative micro-CT analyses of distal femoral metaphysic and middle shaft from wild-type and *Fgfr2* ^S252W/+^ at 2 months and 5 months. (A and C) Three-dimensional images showed reduced trabecular and cortical bone at 2 months, but (B and D) there was more trabecular and cortical bone in mutant mice at 5 months. (E–G, J–M) Quantification of the structural parameters of the femoral metaphysis revealed that Tb.N, Tb.Th, Ct.Th, and Tb.BV/TV, Ct.BV/TV, Tb.BMD, and Ct.BMD were lower than in control mice, Tb.Sp and Tb.SMI were increased at 2 months in mutant mice relative to wild-type mice (*n* = 6)( H and I). (M) Although Ct.BMD levels were less at 5 months in mutant mice than in wild-type mice, Tb.N, Tb.Th, Ct.Th, and Tb.BV/TV, Ct.BV/TV, Tb.BMD levels were higher in mutant mice(E–G and J–L). (H and I) Tb.Sp and Tb.SMI were significantly less pronounced at 5 months in mutant mice than in wild-type mice (*n* = 6). Graphs show mean value ±SD (Student's *t*-test, *P<0.05, **P<0.01).

Consistent with previous results [22], [23], *Fgfr2* ^S252W/+^ mice were shorter than wild type mice and their skull displayed synostosis of the coronal suture as well as other abnormalities (Fig. S1A-C and Table S1). To determine the mass of trabecular bone, micro-computed tomography (micro-CT) analysis of the distal metaphysis of femurs derived from 2-month-old and 5-month-old mice was performed. Three-dimensional images of the femoral metaphysis are shown in Fig. 1A–D. Quantification of the structural parameters revealed that bone trabecular number (Tb.N), trabecular thickness (Tb.Th), cortical thickness (Ct.Th), trabecular and cortical volume/tissue volume (%, Tb.BV/TV, Ct.BV/TV), and trabecular and cortical bone mineral density (Tb.BMD, Ct. BMD) were all lower in mutant mice, by 15.2%, 13.1%, 17.6%, 30.6%, 18.4%, 11.0%, and 4.5%, respectively, at 2 months (Fig. 1E–G and J–M). Trabecular separation (Tb.Sp) in mutant mice was increased by 25.3% (Fig. 1H). These differences in trabecular connectivity of *Fgfr2* ^S252W/+^ femora were confirmed by an increase in trabecular structure model index (Tb.SMI; Fig. 1I). Although cortical bone mineral density (Ct.BMD) was lower in 5 months mutant mice than in controls by 2.1% (Fig. 1M), bone trabecular number (Tb.N), trabecular thickness (Tb.Th), cortical thickness (Ct.Th), trabecular and cortical volume/tissue volume (%, Tb.BV/TV, Ct.BV/TV), and trabecular bone mineral density (Tb.BMD) all had higher values in mutant mice than in controls, by 17.1%, 26.5% (*P<0.05*), 2.1%, 2.4%, and 11.7% (*P<0.05*), respectively, at 5 months in mutant mice (Fig. 1E–G and J–L). Trabecular separation (Tb.Sp) was 45.7% (*P<0.05*) lower in mutant mice (Fig. 1H). These differences were confirmed by a significantly decrease in trabecular structure model index (Tb.SMI; Fig. 1I). Collectively, these observations indicate that gain-of-function mutation in FGFR2 changed bone mass and compromised architecture in adult mice in an age-dependent manner.

### Altered bone formation in Fgfr2S252W/+ mice

**Figure 2:**
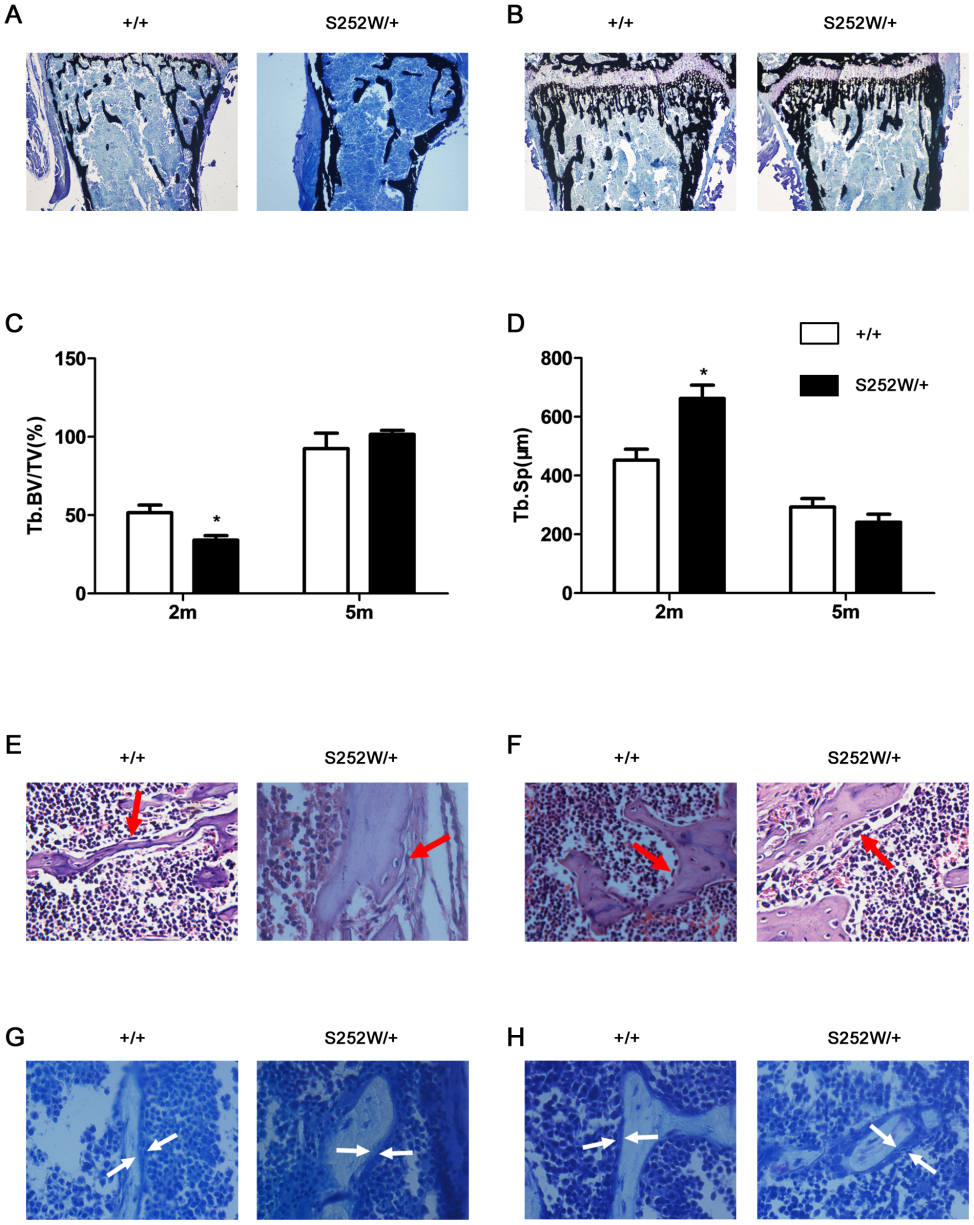
Histochemical analysis of decalcified and undecalcified tibiae from 2- and 5-month-old mutant and wild-type mice. (A and B) Von Kossa staining of undecalcified tibiae revealed that the proximal tibiae of *Fgfr2* ^S252W/+^ mice had sparser trabecular bone than wild-type mice at 2 months, but they had longer and denser trabecular bone than wild-type mice at 5 months (magnification, ×40). (C and D) Histomorphometric measurements of tibiae. The tibiae from *Fgfr2* ^S252W/+^ mice had significantly less Tb.BV/TV and more Tb.Sp at 2 months, but they had more Tb.BV/TV and less Tb.Sp at 5 months (n = 6). (E and F) H&E staining of tibiae showed the morphology of metaphyseal osteoblasts in the proximal tibia. Note that there were fewer osteoblasts lining trabecular bone in the *Fgfr2* ^S252W/+^ mice at 2 months but they were more plump and cuboidal at 5 months (red arrows; magnification, ×400). (G and H) Von Kossa staining showed that osteoids laid down in the trabecular bone in *Fgfr2* ^S252W/+^ mice were thinner than those of wild-type mice at 2 months old, but thicker than those of wild-type mice at 5 months (white arrows; magnification, ×400). Graphs show mean value ±SD (Student's *t*-test, *P<0.05).

To determine whether the changed bone mass in Fgfr2S252W/+ mice was due to altered bone formation, a histologic analysis of decalcified and undecalcified tibiae was performed. Von Kossa staining of undecalcified tibiae showed that the trabecular bone of *Fgfr2* ^S252W/+^ mice was sparser than wild-type at age 2 months but longer and denser than wild-type at 5 months (Fig. 2A and B). The histomorphometric parameters of bone formation in the proximal tibiae are shown in Fig. 2C and D. The Tb.BV/TV in *Fgfr2* ^S252W/+^ were significantly reduced at 2 months (*P<0.05*), but were increased at 5 months (Fig. 2C). Tb.Sp was significantly increased in mutant mice at 2 months (*P<0.05*), but were reduced at 5 months (Fig. 2D). These results are consistent with the results obtained by micro-CT examination. Although the osteoblasts lining trabecular bone of *Fgfr2* ^S252W/+^ mice at 2 months were longer than those in wild-type mice, the osteoblasts were less(Fig. 2E, arrows), which suggests that the osteoblast activity in *Fgfr2* ^S252W/+^ mice was decreased. The osteoblasts lining trabecular bone of *Fgfr2* ^S252W/+^ mice at 5 months were plump and cuboidal than those in wild-type mice (Fig. 2F, arrows), which suggests that the osteoblast activity in *Fgfr2* ^S252W/+^ mice was increased. In *Fgfr2* ^S252W/+^ mice, the trabecular bone was lined with thinner osteoids compared with wild-type mice at 2 months (Fig. 2G, arrows), and it was lined with thicker osteoids than wild-type mice at 5 months old (Fig. 2H, arrows). The increased thickness of osteoid in *Fgfr2* ^S252W/+^ mice suggests that there may be defects in bone matrix mineralization in *Fgfr2* ^S252W/+^ mice. All these bone phenotypes indicate that *Fgfr2* ^S252W/+^ mice have osteomalacia-like skeleton, which need to be further studied.

### Serum levels of Ca, phosphate and PINP in 2 month old and 5 month old Fgfr2^S252W/+^ mice

**Figure 3:**
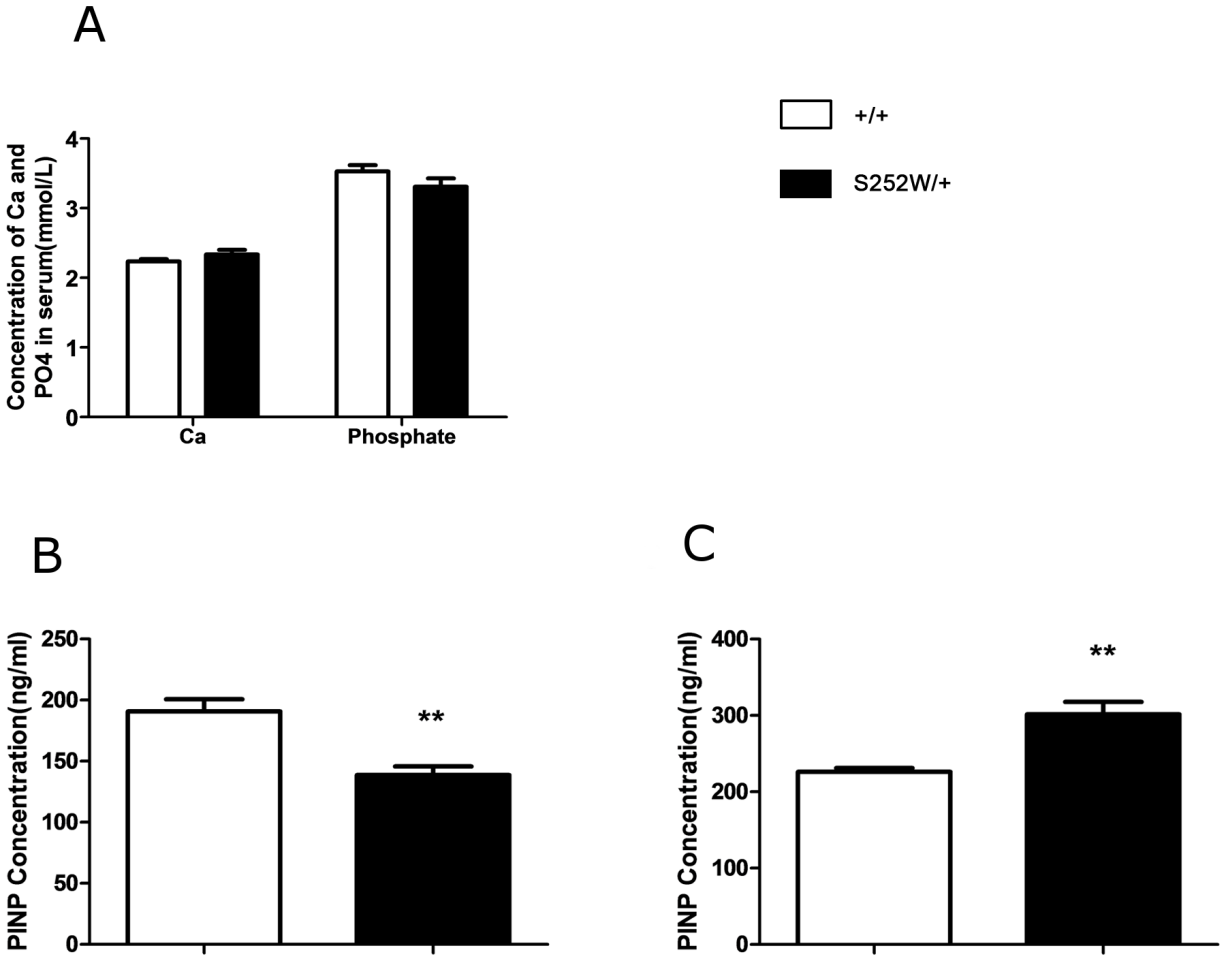
Serum biochemistry and serum level of PINP of wild-type and *Fgfr2* ^S252W/+^mice at 2 months and 5 months. (A) There was no remarkable difference in total serum Ca or phosphate between wild-type and mutant mice at 2 months or at 5 months. (*n* = 6). (B) The serum levels of PINP as measured by ELISA were significantly lower in mutant mice at 2 months than in wild-type mice at 2 months. (C) These levels were significantly higher in mutant mice at 5 months than in wild-type mice at 5 months (*n* = 6). Graphs show mean value ±SD (Student's *t*-test, **P<0.01).

To determine whether bone defects observed in *Fgfr2* ^S252W/+^ mice are related to systemic alterations in mineral homeostasis, levels of total Ca and phosphate in mouse serum were measured. As shown in Fig. 3A, no significant differences in serum levels of either total Ca or phosphate were found between control and mutant mice. Procollagen I N-Terminal Propeptide (PINP) is a sensitive and specific marker of osteoblast activity. Serum levels of PINP were detected using ELISA and were found to be lower than in controls at 2 months (Fig. 3B) but higher than in controls at 5 months in *Fgfr2* ^S252W/+^ mice (Fig. 3C), suggesting less osteoblast activity in *Fgfr2* ^S252W/+^ mice than in controls at 2 months but more at 5 months.

### S252W mutation of FGFR2 represses the proliferation and adipogenic differentiation of BMSCs

**Figure 4:**
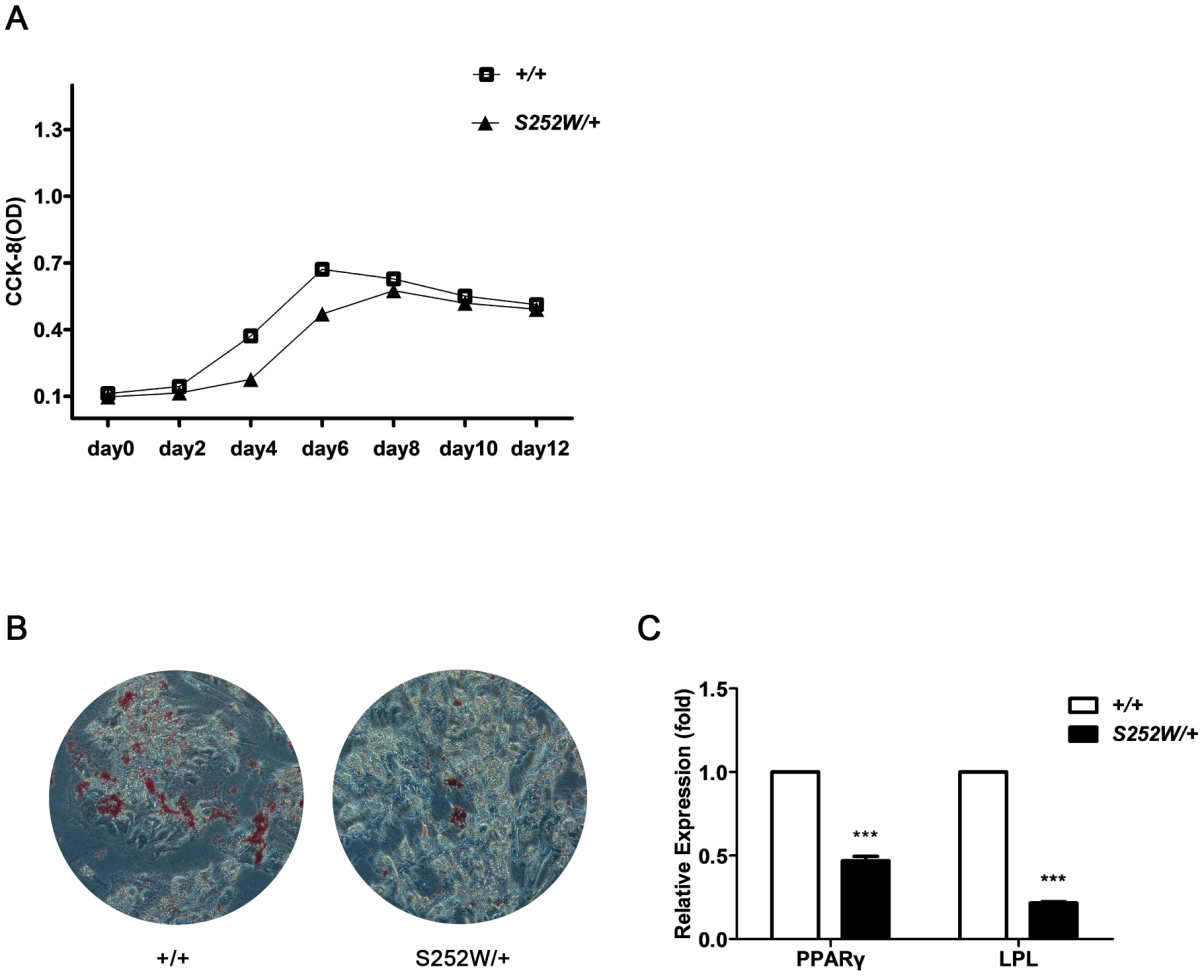
S252W mutation affected both proliferation and adipogenic differentiation of BMSCs. (A) CCK-8 assay showed that *Fgfr2* ^S252W/+^ BMSCs proliferated more slowly than wild-type BMSCs. As indicated by the absorbance values, 2 days after inoculation, there was little increase in cell number of wild-type BMSCs; on day 4, there were significantly more cells; on day 6, the number of cells peaked and cell proliferation entered a stationary phase. The number of *Fgfr2* ^S252W/+^ BMSCs did not change significantly during the first 4 days after inoculation. The first significant increase was observed on day 6 and the number of cells peaked on day 8. All growth phases of *Fgfr2* ^S252W/+^ BMSCs lagged behind those of wild-type BMSCs. (B) After 21 days of adipogenic induction, lipid droplets were stained positive by oil red O in both *Fgfr2* ^S252W/+^ BMSCs cultures and with wild-type BMSCs cultures, but *Fgfr2* ^S252W/+^ BMSCs produced significantly fewer lipid droplets than wild-type BMSCs. (C) qRT-PCR detection of adipogenesis-related genes PPARγ and LPL. The results showed that adipogenic induction induced significantly less expression of PPARγ and LPL in *Fgfr2* ^S252W/+^ BMSCs than in wild-type BMSCs. Graphs show the mean value ±SD (Student's t-test, *** *P*<0.001).

This age-dependent bone phenotype indicated defective bone homeostasis in *Fgfr2* ^S252W/+^ mice. Adult bone homeostasis depends on BMSCs that give rise to osteoblasts, adipocytes, chondrocytes, and other types of cells [31], [32]. BMSCs from *Fgfr2* ^S252W/+^ mice exhibited less proliferative capacity than cells from wild-type mice (Fig. 4A). Lipid droplets were detected using oil red O staining in cells from both wild-type and mutant mice after 21 days of adipogenic induction. Cells from *Fgfr2* ^S252W/+^ mice had fewer lipid droplets than cell from wild-type mice (Fig. 4B). qRT-PCR was used to detect the expression of the adipogenesis-related genes peroxisome proliferator-activated receptor-γ (PPARγ) and lipoprotein lipase (LPL). Because the adipogenic differentiation of BMSCs from *Fgfr2* ^S252W/+^ mice was suppressed, expression levels of PPARγ and LPL were significantly lower in cells from mutant mice than in those from wild-type mice (*P*<0.001) after adipogenic induction (Fig. 4C).

### S252W mutation of FGFR2 affects osteogenic differentiation of BMSCs and bone matrix mineralization of osteoblasts

**Figure 5:**
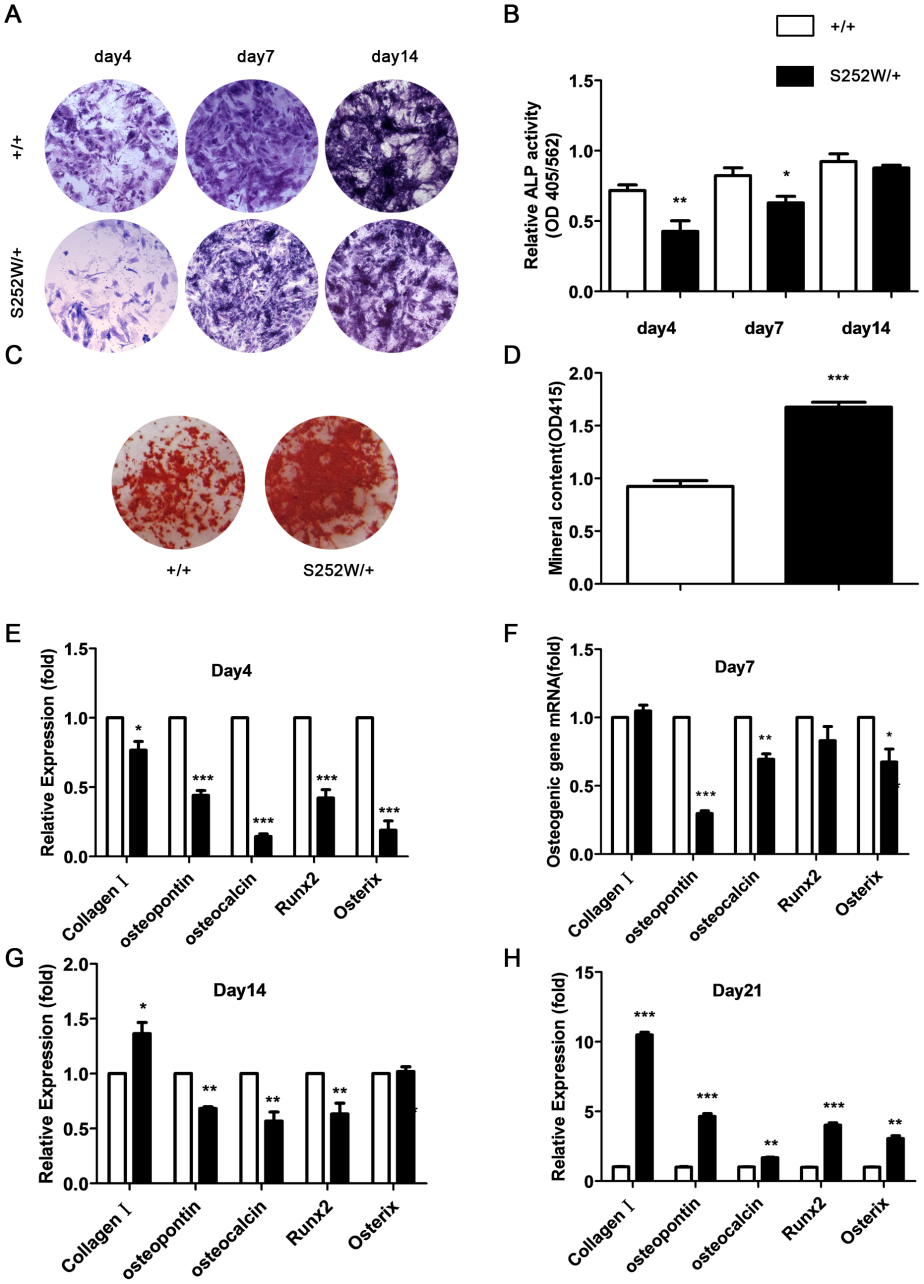
Effects of S252W mutation on the osteogenic differentiation and mineralization of BMSCs. (A) ALP staining showed less crystal violet-staining cells in cultured *Fgfr2* ^S252W/+^ BMSCs than in wild-type BMSCs on days 4, 7, and 14. (B) There was significantly less ALP activity (normalized to the total protein content of the sample, 562 nm) in *Fgfr2* ^S252W/+^ BMSCs. (C) Alizarin red staining of the mineralized osteoblasts showed more mineralized nodules on day 21 after osteogenic differentiation in *Fgfr2* ^S252W/+^ mice. (D) Bound alizarin red was dissolved with 5% SDS, 0.5 N HCl and measured at 415 nm to quantify the mineral content. Cultured *Fgfr2* ^S252W/+^ BMSCs showed more mineral content. (E–H) Relative expression levels of osteogenic marker genes were measured using qRT–PCR. The levels of *oc, op*, Runx2 and osteorix mRNA expression in differentiated BMSCs were markedly lower in *Fgfr2* ^S252W/+^ BMSCs on days 4, 7, and 14, but markedly higher on day 21. The expression levels of Col1 mRNA in differentiated BMSCs were markedly lower in *Fgfr2* ^S252W/+^ BMSCs on day 4, but they were higher on day 7 and markedly higher on days 14 and 21. Graphs show mean value ±SD (Student's *t*-test, *P<0.05, **P<0.01, ***P<0.001).

To assess the effects of S252W mutation on the osteogenic differentiation of BMSCs, the ALP activity, the formation of alkaline phosphatase (ALP)-positive colonies and numbers of mineralized bone nodules (Alizarin red staining) were compared in BMSCs isolated from both wild-type and *Fgfr2* ^S252W/+^mice. After 4, 7, 14 days of culture, the BMSCs from *Fgfr2* ^S252W/+^ mice showed fewer crystal-violet-stained cells (Fig. 5A). The ALP activity of BMSCs was significantly lower in *Fgfr2* ^S252W/+^ mice than in wild-type mice (Fig. 5B). On day 21 *Fgfr2* ^S252W/+^ BMSCs had larger mineralized colonies and more absorbance of alizarin red (Fig. 5C and D) (*P*<0.001). These results suggest that gain-of-function mutation of FGFR2 inhibited the osteogenic differentiation of BMSCs in early adulthood but promoted mineralization in old age.

RNA harvested from BMSCs, which were isolated from 6–8-week-old mice, after 4, 7, 14, and 21 days of culture was used to measure the levels of expression of genes related to osteogenic differentiation using qRT-PCR. As shown in Fig. 4E–H, the levels of expression of osteopontin(*op*), osteocalcin(*oc*), Runx2, osterix of BMSCs were all lower in *Fgfr2* ^S252W/+^ mice on days 4, 7, and 14, but were all increased on day 21 compared with wild-type mice. The expression levels of type I collagen (Col1) of BMSCs was lower in *Fgfr2* ^S252W/+^ mice than in wild-type mice on day 4 but higher on days 7, 14, and 21. These results further indicate that gain-of-function mutation of FGFR2 affects osteogenic differentiation of BMSCs in an age-dependent manner.

### Upregulation of SFRP1, SFRP2, and SFRP4 and downregulation of Wnt/β-catenin signaling pathway in S252W BMSCs

**Figure 6:**
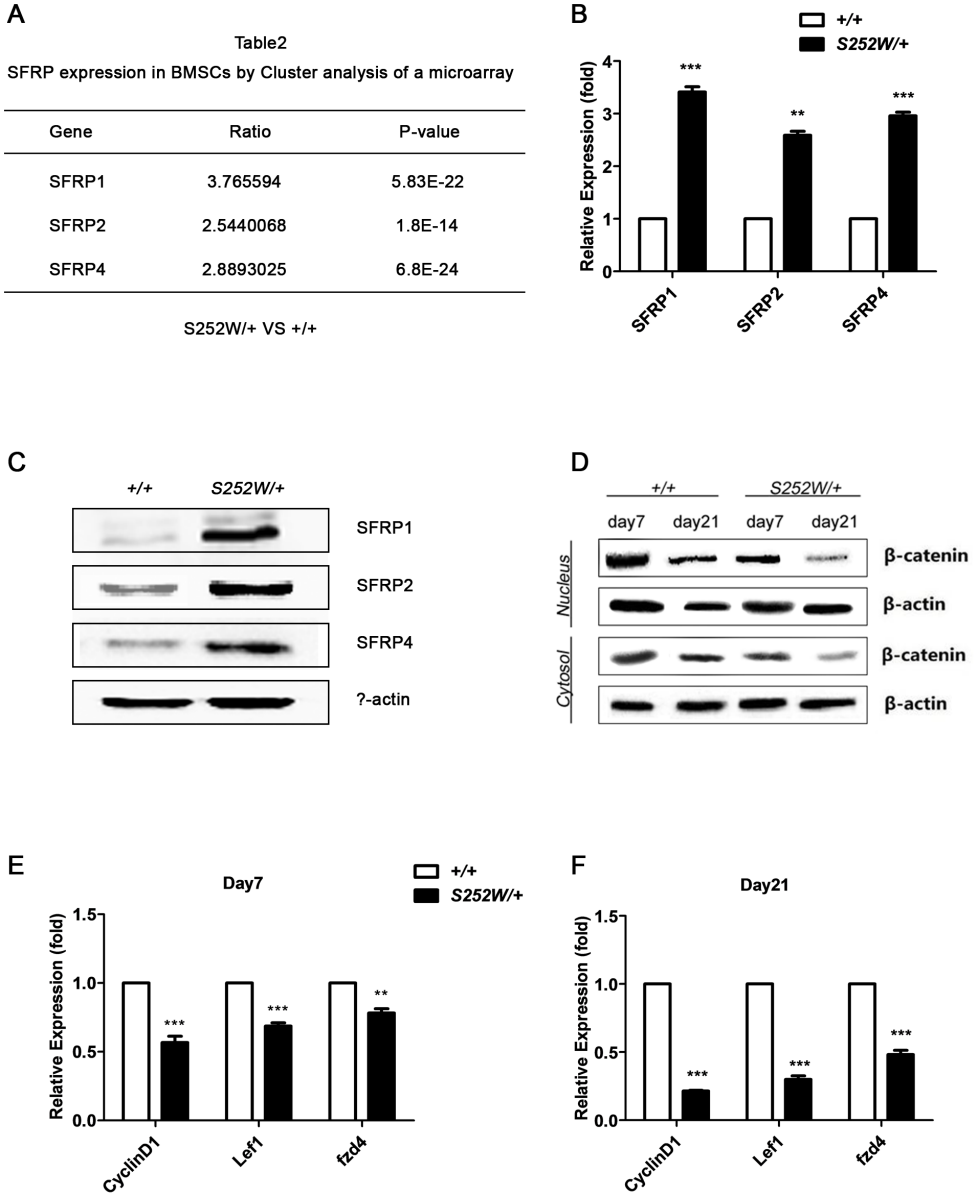
S252W mutation affected Wnt signaling in osteogenically differentiating BMSCs. (A) Cluster analysis of a microarray data revealed that Wnt pathway antagonists SRRP1, SFRP2, and SFRP4 were up-regulated 3.8 fold, 2.5 fold, and 2.9 fold in mutant BMSCs, respectively (*n* = 3). (B) Relative expression levels of SFRP1, SFRP2, and SFRP4 measured by qRT–PCR. The expression levels of SFRP1, SFRP2, SFRP4 mRNA were markedly higher in *Fgfr2* ^S252W/+^ BMSCs. Graphs show mean value ±SD (Student's *t*-test, **P<0.01, ***P<0.001). The PCR results were consistent with microarray results. (C) Western blot analysis demonstrated that the levels of SFRP1, SFRP2, and SFRP4 were higher in *Fgfr2* ^S252W/+^ BMSCs cultured in standard medium. (D) *Fgfr2* ^S252W/+^ BMSCs and wild-type BMSCs were kept in osteogenic differentiation medium. Cells were collected on the indicated days and then cytosol and nuclear levels of Wnt pathway effector β-catenin were examined by Western blot analysis. The results showed that β-catenin protein levels were lower in mutant cells than in wild type cells. (E) Relative expression levels of Wnt target genes *cyclinD1*, *lef1*, and *fzd4* were measured by qRT–PCR. The results showed that after 21 days of osteogenic induction, the mRNA levels of these genes were markedly decreased in *Fgfr2* ^S252W/+^ BMSCs. Graphs show mean value ±SD (Student's *t*-test, **P<0.01, ***P<0.001).

To investigate the mechanism underlying the alterations in osteogenic differentiation of S252W BMSCs, microarray data using cluster analysis was assessed. Wnt pathway antagonists secreted frizzled-related proteins 1, 2, and 4 (SFRP1, SFRP2, and SFRP4) were all up-regulated in BMSCs isolated from *Fgfr2* ^S252W/+^ mice (Fig. 6A). This up-regulation was further confirmed using qRT-PCR (Fig. 6B) (*P*<0.001, *P*<0.01, *P*<0.001) and Western blot using specific antibodies (Fig. 6C). SFRPs are secreted inhibitors of Wnt signaling pathway that act by directly binding to Wnt ligands or frizzled receptors and up-regulation suggested that Wnt signaling might be suppressed in S252W BMSCs [33]. To determine whether canonical Wnt/β-catenin signaling is affected in S252W BMSCs, β-catenin proteins were separated into cytosolic and nuclear fractions and the protein levels were assessed. The results showed that, after 7 or 21 days of osteogenic induction, the cytosolic and nucleus β-catenin levels in *Fgfr2* ^S252W/+^ BMSCs were lower than in the age-matched wild-type BMSCs, indicating suppression of Wnt/β-catenin signaling in mutant cells. In both mutant and wild-type BMSCs, β-catenin protein levels continued to decrease with as culture continued and the decrease was more pronounced in S252W cells (Fig. 6D). To corroborate the conclusion that Wnt pathway was suppressed in mutant cells, the levels of expression of the Wnt target genes *cyclinD1*, *lef1*, and *fzd4* were assessed using qRT-PCR. In keeping with low Wnt signaling in mutant BMSCs, qRT-PCR results showed that their expression levels were reduced (Fig. 6E and F). Expression levels of these genes on osteogenic induction day 21 were lower than on day 7, consistent with levels of β-catenin. Given the critical role of the canonical Wnt/β-catenin pathway in osteogenesis, inhibition of Wnt/β-catenin signaling may underlie the osteogenic defects of *Fgfr2* ^S252W/+^ BMSCs [34].

### Increasing Wnt/β-catenin signaling by Wnt3a treatment ameliorates impaired differentiation of FGFR2 mutant BMSCs

**Figure 7:**
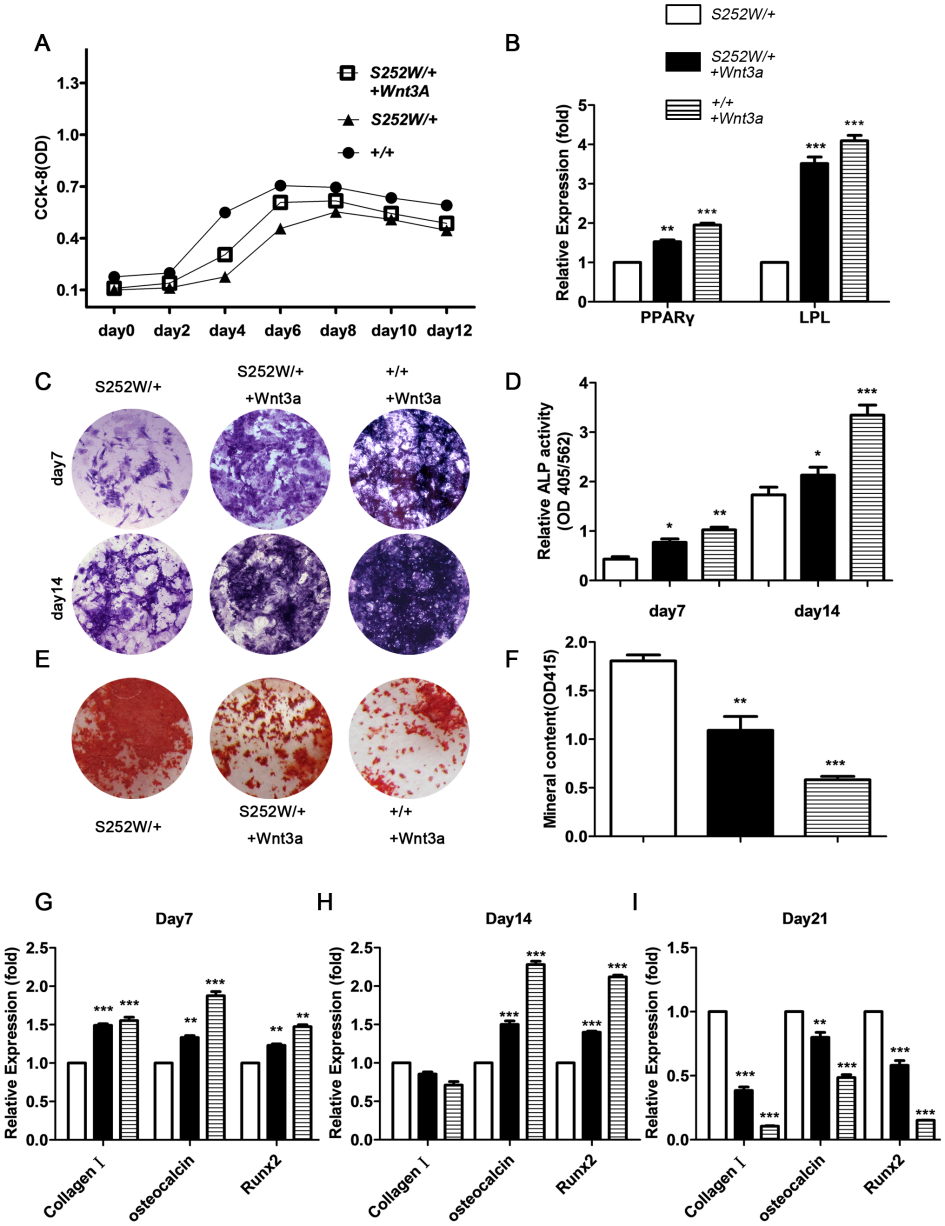
Activated FGFR2 and the proliferation, adipogenic differentiation, osteogenic differentiation, and mineralization of BMSCs. (A) CCK-8 proliferation assay showed *Fgfr2* ^S252W/+^ BMSCs treated with Wnt3a to proliferate less than wild-type BMSCs treated with Wnt3a but more than untreated *Fgfr2* ^S252W/+^. (B) After 21 days of adipogenic induction, expression of adipogenesis-related genes PPARγ and LPL was examined using qRT -PCR. Results showed that expression levels of PPARγ and LPL in *Fgfr2* ^S252W/+^ BMSCs treated with Wnt3a were lower than in wild-type+Wnt3a BMSCs but significantly higher than in untreated *Fgfr2* ^S252W/+^ BMSCs. (C) ALP staining showed more crystal violet-staining cells in cultured *Fgfr2* ^S252W/+^ treated with Wnt3a BMSCs than in untreated *Fgfr2* ^S252W/+^ BMSCs on days 7 and 14. (D) ALP activity (normalized to the total protein content of the sample, 562 nm) was significantly higher in *Fgfr2* ^S252W/+^ treated with Wnt3a BMSCs than in untreated cells. (E) After 21 days of osteogenic differentiation, Alizarin red staining of the mineralized osteoblasts showed that wild-type BMSCs treated with Wnt3a had the fewest mineralized nodules, followed by *Fgfr2* ^S252W/+^ treated with Wnt3a, and untreated *Fgfr2* ^S252W/+^ BMSCs had the most nodules. (F) Cultured wild-type BMSCs treated with Wnt3a showed the least mineral content, followed by *Fgfr2* ^S252W/+^ BMSCs treated with Wnt3a, and untreated *Fgfr2* ^S252W/+^ BMSCs had the highest mineral content. (G) Relative expression levels of osteogenic marker genes were measured using qRT–PCR. The expression levels of *oc*, and Runx2 mRNA were markedly higher in differentiated *Fgfr2* ^S252W/+^ treated with Wnt3a BMSCs than in untreated *Fgfr2* ^S252W/+^ BMSCs on days 7 and 14, but markedly lower on day 21. The expression levels of Col1 were markedly higher in *Fgfr2* ^S252W/+^ BMSCs treated with Wnt3a than in untreated *Fgfr2* ^S252W/+^ BMSCs on days 7, but lower on day 14, 21. Graphs show mean value ±SD (Student's *t*-test, *P<0.05, **P<0.01, ***P<0.001).

If osteogenic differentiation defects in *Fgfr2* ^S252W/+^ BMSCs are caused by impairment of the Wnt pathway, forced increases in Wnt signaling may be able to mitigate the phenotype. To activate the Wnt/β-catenin signaling pathway, 100 ng/mL of Wnt3a was added to the cell culture medium. β-catenin protein levels in both the cytoplasm and nucleus of *Fgfr2* ^S252W/+^ BMSCs were increased after adding Wnt3a to osteogenic medium. The proliferative, adipogenic, and osteogenic capabilities of *Fgfr2* ^S252W/+^ BMSCs and wild-type BMSCs were examined after Wnt3a treatment. Wnt3a treatment significantly enhanced proliferation of *Fgfr2* ^S252W/+^ BMSCs and wild-type BMSCs (Fig. 7A). The expression levels of the two adipogenesis-related genes PPARγ and LPL were examined after 21 days of adipogenic induction. The results showed that adding Wnt3a to adipogenic induction medium can markedly enhance expression of PPARγ and LPL in *Fgfr2* ^S252W/+^ BMSCs (Fig. 7B) (*P*<0.01, *P*<0.001). Results also showed that adding Wnt3a to osteogenic medium can promote osteogenic capacity of *Fgfr2* ^S252W/+^ BMSCs as evidenced by ALP staining of cells induced on day 7 and day 21 (Fig. 7C). ALP activity analysis confirmed an increased activity after Wnt3a treatment (Fig. 7D) (*P*<0.05). However, there was significantly less mineralization after 21 days of Wnt3a treatment in *Fgfr2* ^S252W/+^ BMSCs (Fig. 7E, F) (*P*<0.01). In line with the decrease in mineralization, the expression levels of osteocyte-related genes Col1, *oc*, Runx2 in *Fgfr2* ^S252W/+^ BMSCs were significantly lower after 21 days of osteogenic induction in the presence of Wnt3a (Fig. 7G) (*P*<0.001, *P*<0.01, *P*<0.001). In this way, reduced differentiation of BMSCs was found to be largely due to inhibition of Wnt signaling.

## Discussion

Skeletal defects caused by Apert syndrome mutations during the fetal and childhood development have been well studied, but the effects of these mutations on adult bone homeostasis have been less frequently addressed. The present study, which involved an adult Apert syndrome mouse model, revealed an age-dependent bone phenotype caused by S252W mutation of FGFR2. In early adult stages, *Fgfr2* ^S252W/+^ mutant mice have markedly less bone mass than wild-type mice. This is consistent with the results reported by Zhou et al. (2013). Like humans, mice exhibit an age-related bone loss. They have been used to model osteoporosis [35]. Unlike wild-type mice, whose bone mass decreases with age, mutant mice did not show any age-related bone loss. Instead, their bone mass increased (Fig. 1A–D). These results show that this bone phenotype of mutant mice was not caused by systemic alteration in mineral homeostasis but was instead the result of increased osteoblast activity in adult mice. Bone homeostasis in adults involves the progeny of a small number of cells called mesenchymal stem cells (MSCs), which give rise to osteoblasts, adipocytes, chondrocytes, and a few other types of cells [31], [32]. Osteoblasts and adipocytes can further differentiate into bone mass and fat mass, respectively [32]. It has been hypothesized that enhanced differentiation into either the osteoblastic or adipocytic lineages occurs at the expense of the alternative lineage [36], [37]. To determine whether higher bone mass in 5 month old mutant mice is the outcome of altered lineage commitment of BMSCs, *Fgfr2* ^S252W/+^ BMSCs were isolated and their osteogenic and adipogenic differentiation were both found to be suppressed. Unlike ossification, which is carried out by BMSCs-derived osteoblasts, bone mass also depends on an opposite process, termed bone resorption, which is performed by macrophage-derived osteoclasts [38]. In this way, the phenotype of lower bone mass in young adult mice is, at least in part, a result of suppression in lineage commitments of BMSCs, and the higher bone mass in older adult mice might be at least partially due to altered BMSC differentiation.

Microarray analysis revealed upregulation of extracellular Wnt signaling antagonists SFRP1/2/4 in *Fgfr2* ^S252W/+^ BMSCs. SFRPs are secreted inhibitors of Wnt signaling that act by directly binding to Wnt ligands or Frizzled receptors [33]. The pronounced expression of these SFRPs in *Fgfr2* ^S252W/+^ BMSCs suggested a down-regulation of Wnt signaling. Wnt/β-catenin signaling was suppressed in *Fgfr2* ^S252W/+^ BMSCs as indicated by lower levels of β-catenin protein. The Wnt pathway is a critical mechanism underlying the regulation of skeletal development and bone homeostasis [34]. Canonical Wnt/β-catenin signaling represses BMSCs' commitment to the chondrogenic and adipogenic lineages and enhances commitment to osteoblastic lineage [34]. The importance of the Wnt pathway in bone homeostasis is further underlined by the isolation of bone disease-related mutations of Wnt co-receptor *LRP5*, with loss-of-function mutations in *LRP5* associated with low bone mass in osteoporosis-pseudoglioma syndrome whereas gain-of-function was found to be associated with high bone mass [39]–[41]. Because they are antagonists of Wnt signaling, SFRPs also play important roles in bone homeostasis. Deletion of the SFRP1 gene leads to increased bone mass [42]. Over-expression of SFRP1 or SFRP4 caused loss of bone mass [43]–[45]. Given the importance of Wnt pathway in bone formation, the strong correlations of elevation in SFRP1/2/4 protein levels and low canonical Wnt signaling in early BMSCs with low bone mass in young adult *Fgfr2* ^S252W/+^ mice suggested that down-regulation of Wnt/β-catenin might be one of mechanisms underlying bone loss in Apert patients. Osteoblasts expressing *Fgfr2* ^S252W/+^ showed a striking decrease in expression of many Wnt target genes [46]. Mansukhani et al. [46] further showed that expression of FGFR2 activation mutations S252W or C342Y stimulated expression of the transcription factor Sox2, which is associated with β-catenin to suppress expression of many Wnt target genes. Upstream Wnt signaling was also impaired, most likely due to enhanced expression of Wnt antagonists SFRP1/2/4 in *Fgfr2* ^S252W/+^ mice. The dysregulation of Wnt signaling was found to be responsible for the defective proliferation and differentiation of BMSCs, and all these defects could be reversed by adding Wnt3a. In this way, the present study unraveled another layer of regulation by which S252W mutation can directly suppress the upstream Wnt signaling through up-regulation of Wnt inhibitors SFRPs. The combinatorial inhibition of Wnt signaling both upstream and downstream underlines the pathogenesis of Apert syndrome.

The phenotype of increased bone mass in 5 month old mice was not as expected. However, Zhou et al. [47] showed that abnormalities in bone architecture of *Fgfr2* ^S252W/+^ mice became less severe with age, probably due to catch-up growth between P28 and P56 [47]. This catch-up growth most likely continued beyond P56, resulting in an accumulation of bone mass in 5 month old mice. Given the pivotal role of Wnt pathway in promoting osteogenic differentiation of BMSCs, it is paradoxical that low Wnt signaling in BMSCs and high bone mass would coexist in *Fgfr2* ^S252W/+^ mice. It has been reported that Wnt signaling can stimulate differentiation of early osteoblasts but inhibits the mineralization of mature osteoblasts [48]. In this way, it is conceivable that low Wnt signaling may enhance mineralization in mature osteoblasts, leading to high bone mass in *Fgfr2* ^S252W/+^ mice. However, low Wnt signaling, which can be due to over-expression of *SFRP1* [46] or *SFRP4* [44], [45] or to loss-of-function mutations in *LRP5* [40]–[42], has never been found to be associated with high bone mass in other mouse models. One explanation for this is that Wnt signaling may be less suppressed in *Fgfr2* ^S252W/+^ mice than in the aforementioned *SFRP* overexpressing or *LRP5* mutation mice. This may lead to less inhibition of osteogenic differentiation, which may provide sufficient osteoblasts to support catch-up growth. During the catch-up growth phase, low levels of Wnt signaling may promote mineralization of mature osteoblasts, causing a high-bone-mass phenotype. Another explanation is that *Fgfr2* ^S252W/+^ mice with this abnormal bone phenotype could be an outcome of combinatorial effects of malfunctions of multiple pathways. Other pathways, including the MAPK pathway, PKC pathway, and AKT pathway were all found to be dysregulated in *Fgfr2* ^S252W/+^ mice.

Taken together, the present study revealed an age-related bone mass phenotype in *Fgfr2* ^S252W/+^ mice and provided evidence to show that dysregulation of Wnt signaling in both BMSCs and osteoblasts plays an important role in defective ossification in *Fgfr2* ^S252W/+^ mice, so shedding light on the mechanism underlying Apert syndrome.

## Supporting Information

Figure S1 A) Skulls of *Fgfr2* ^S252W/+^ mice have abnormalities resembling those observed in human Apert syndrome. As revealed by Micro-CT, the skull of the mutant mouse shows extreme brachycephaly, extreme reduction anteroposteriorly of the frontal bones, and severe mandibular prognathism. The red arrow indicates the synostosis of the coronal suture, and the yellow arrow indicates the delayed fusion of the interfrontal suture. (B and C)*Fgfr2* ^S252W/+^ mice at 2 months had a smaller body size and short limbs. (D) Gene expression results of bone marrow mesenchymal stem cells (BMSCs). Histogram of fold changes of gene expression: Gene expression of BMSCs harvested from 6- to 8-week-old FGFR2 mutant mice and their wild-type littermates was evaluated using a microarray. Results showed that, in mutant cells, the expression of 408 genes had changed. This included 327 upregulated genes and 81 down-regulated genes. Cluster analysis: Clustering was performed to visualize the correlations among the replicates under different sample conditions. Up- and down-regulated genes are indicated in red and green, respectively. A subset of differential genes was selected for clustering analysis. An intensity filter was used to select genes where the difference between the maximum and minimum intensity values exceeds 300 among all microarrays. For this microarray project, the number of genes clustered was 277. Results showed that 107 genes had their expression levels up-regulated at least 2 fold.
(TIF)

Table S1 **Abnormalities in Phenotype of** ***Fgfr2*** **^S252W/+^ mice.**
(DOC)
