# Supplementary figures and images for: Inhibited Wnt Signaling Causes Age-Dependent Abnormalities in the Bone Matrix Mineralization in the Apert Syndrome FGFR2S252W/+ Mice
Source: PLoS One. 2015 Feb 18;10(2):e112716. doi: 10.1371/journal.pone.0112716 (PMC4333342; doi:10.1371/journal.pone.0112716)

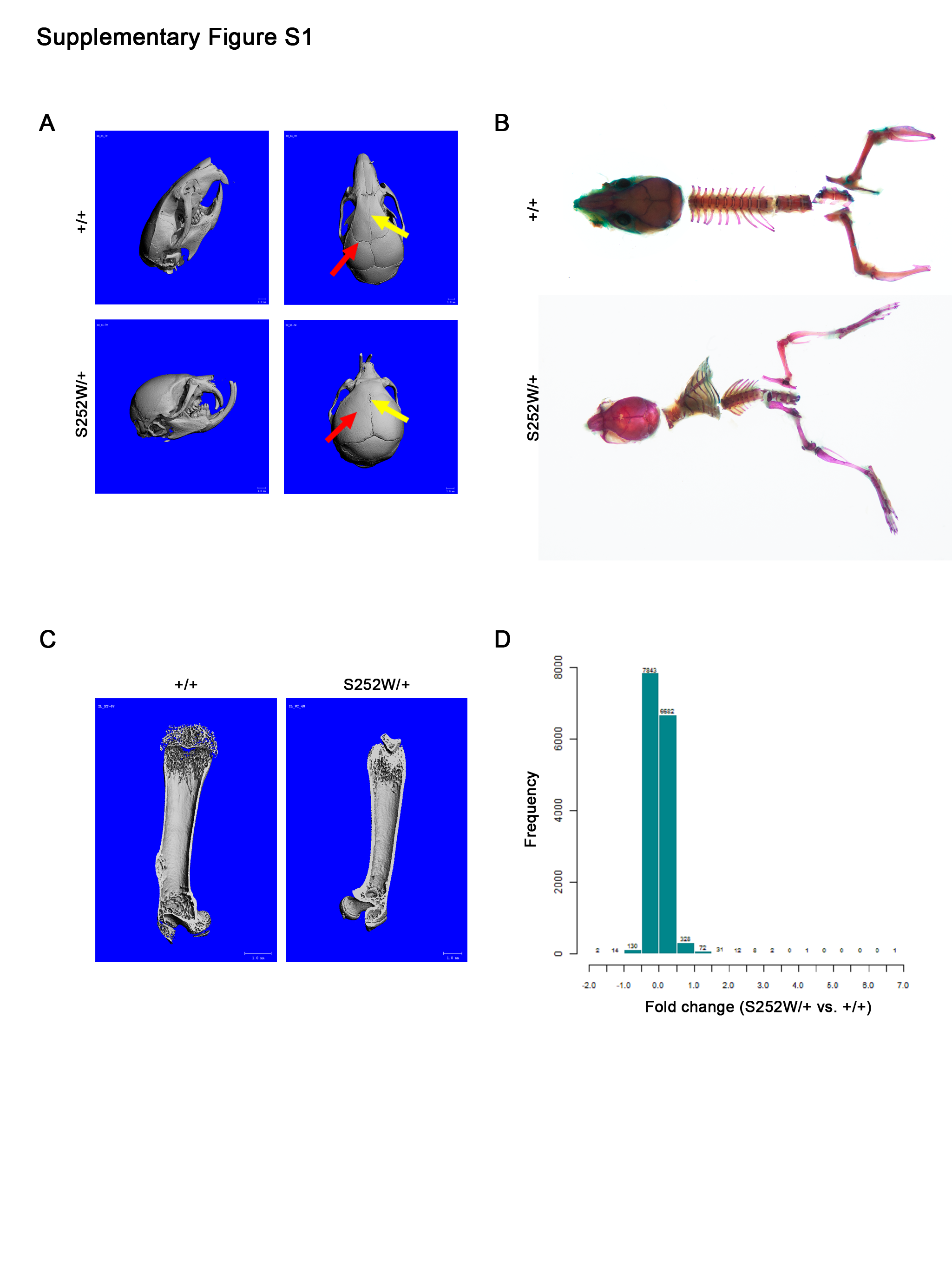

Supplement: Figure S1 — A) Skulls of Fgfr2 S252W/+ mice have abnormalities resembling those observed in human Apert syndrome. As revealed by Micro-CT, the skull of the mutant mouse shows extreme brachycephaly, extreme reduction anteroposteriorly of the frontal bones, and severe mandibular prognathism. The red arrow indicates the synostosis of the coronal suture, and the yellow arrow indicates the delayed fusion of the interfrontal suture. (B and C)Fgfr2 S252W/+ mice at 2 months had a smaller body size and short limbs. (D) Gene expression results of bone marrow mesenchymal stem cells (BMSCs). Histogram of fold changes of gene expression: Gene expression of BMSCs harvested from 6- to 8-week-old FGFR2 mutant mice and their wild-type littermates was evaluated using a microarray. Results showed that, in mutant cells, the expression of 408 genes had changed. This included 327 upregulated genes and 81 down-regulated genes. Cluster analysis: Clustering was performed to visualize the correlations among the replicates under different sample conditions. Up- and down-regulated genes are indicated in red and green, respectively. A subset of differential genes was selected for clustering analysis. An intensity filter was used to select genes where the difference between the maximum and minimum intensity values exceeds 300 among all microarrays. For this microarray project, the number of genes clustered was 277. Results showed that 107 genes had their expression levels up-regulated at least 2 fold. (TIF) [file pone.0112716.s001.tif]
