# Supplementary material for: Inhibited Wnt Signaling Causes Age-Dependent Abnormalities in the Bone Matrix Mineralization in the Apert Syndrome FGFR2S252W/+ Mice
Source: PLoS One. 2015 Feb 18;10(2):e112716. doi: 10.1371/journal.pone.0112716 (PMC4333342; doi:10.1371/journal.pone.0112716)
Supplement: Table S1 — Abnormalities in Phenotype of Fgfr2 S252W/+ mice. (DOC) [file pone.0112716.s002.doc]

**Table S1. Abnormalities in Phenotype of *Fgfr2*^S252W/+^ mice**

| **Phenotype** | **Time** | **Wild type mice** | ***Fgfr2*^S252W/+^ mice** |
| --- | --- | --- | --- |
| Body size/ weight | At birth | 100%/100% | 93%/90% |
|  | 3 weeks | 100%/100% | 60-72%/40-50% |
|  | 3 months | 100%/100% | 80-85%/65-70% |
| Body fat | 2 months | Normal | Normal |
|  | 5 months | Normal | Lean |
| Head | At birth | Normal | Domed |
| Eyes |  | Normal | Widely spaced |
| Face | At birth | Normal | Short |
| Teeth |  | Normal | Long incisor teeth |
| Occlusion |  | Normal | Malocclusion with age |
| The palate |  | Normal | Malformation |
| Nasal cartilage |  | Normal | Thickened |
| The basicranium |  | Normal | Increased cartilage |
| The sternum |  | Normal | Fusion |
| The trachea |  | Normal | Complete cartilage sleeve |
| The zygomatic arch bones |  | Normal | Fusion of joints separating |

Note: In addition to premature fusion of the cranial sutures, severe syndactyly, the mutant mice show other abnormalities in internal organs.

[1] Holmes G, Rothschild G, Roy UB, Deng CX, Mansukhani A, et al. (2009) Early onset of craniosynostosis in an Apert mouse model reveals critical features of this pathology. Dev Biol 328: 273–284.

# [2] Chen P, Zhang L, Weng T, Zhang S, Sun S, et al. (2014) A Ser252Trp mutation in fibroblast growth factor receptor 2 (FGFR2) mimicking human Apert syndrome reveals anessential role for FGF signaling in the regulation of endochondral bone formation. Plos one 9:e87311.

[3] Zhou X, Pu D, Liu R, Li X, Wen X, et al.(2013)The Fgfr2(S252W/+) mutation in mice retards mandible formation and reduces bone mass as in human Apert syndrome . Am J Med Genet A 161:983-992.

[4] Yingli Wang, Ran Xiao, Fan Yang, Baktiar O, Karim, et al. (2005)Abnormalities in cartilage and bone development in the Apert syndrome FGFR2+/S252W mouse. Development 132: 3537–3548.

[5] Chen L, Li D, Li C, Engel A, Deng CX (2003) A Ser250Trp substitution in mouse fibroblast growth factor receptor 2 (Fgfr2) results in craniosynostosis*.* Bone 33: 169–178.
